# Exploration of the food environment in different socioeconomic areas in Hong Kong and Singapore: a cross-sectional case study

**DOI:** 10.1186/s12889-023-15953-9

**Published:** 2023-06-13

**Authors:** Ivan Ho, Tricia Chng, Sue Kleve, Tammie Choi, Julie Brimblecombe

**Affiliations:** Department of Nutrition, Dietetics and Food, School of Clinical Sciences, Monash University, Melbourne, Vic 3168 Australia

**Keywords:** Public health, Mapping, Food outlets, Singapore, Hong Kong, Socioeconomic status

## Abstract

This study explored the local food environment of Hong Kong and Singapore using a qualitative case study approach to inform future upstream public health nutrition policies. Food outlets that provide food to be eaten in the home were mapped in selected areas of high and low socioeconomic status (SES) of Hong Kong and Singapore. Food outlet density relative to land area was determined. In both countries, lower SES areas surveyed were shown to have higher food outlet density while higher SES areas had fewer but larger food outlets. In Hong Kong, both SES areas reported similar proportions of healthy and unhealthy food outlets.

This study highlights the accessibility of food outlet types through their geographical location and density. Future research assessing the differences in eating culture between these two countries should be considered alongside this study’s findings, to investigate strategies influencing the food environment in order to promote healthier eating habits.

**Supplementary Information:**

The online version contains supplementary material available at 10.1186/s12889-023-15953-9.

## Background

Escalating rates of obesity and associated co-morbidities globally have become a dominant concern in the field of public health nutrition. The Socio-ecological Model [1] indicates that individual behaviour such as diet is complex and can be influenced by the environment at different levels. In recent years, there has been increasing acknowledgement of the food environment as a major influence on population diet [2]. Numerous organisations such as the Food and Agriculture Organisation (FAO) and World Health Organisation (WHO) have incorporated recommendations within their guidelines in order to influence the food environment to positively impact population health [2]. Some of these recommendations include reducing the cost of nutritious foods and improving food storage and market infrastructure [2].

The FAO defines the food environment as ‘the foods available to people in their surroundings as they go about their everyday lives and the nutritional quality, safety, price, convenience, labelling and promotion of these foods’ [3] while the European Public Health Alliance (EPHA) defines the food environment as ‘a combination of the ‘spaces’ in which people make decisions about food, and the foods and drinks that are made available, accessible, affordable and desirable in those spaces’ [4]. For the purpose of this study, the food environment is referred to as the food and drinks that are available, accessible, affordable and desirable to people in the spaces where people make decisions about what to eat based on their nutritional quality, safety, price, convenience, labelling and promotion.

A combination of the definitions by FAO and EPHA is used to inform the food environment definition in this paper to acknowledge the various components of the food environment and their impact on the decisions people make about food.

Glanz et al. further categorised the food environment into community and consumer nutritional environments [5]. The community nutritional environment is defined as the local foodscape which affects population dietary options and choices through the number, type, location and accessibility of food outlets in a designated area. The consumer nutritional environment is defined as the environment within a food outlet in which the consumer is exposed to; which takes into account food cost, availability, placement and quality [5].

A number of studies that have focused on the community nutritional environment have examined the influence of food outlets in an area and its relationship with population diet [6–10]. Evidence indicate that people living within close proximity to healthy food outlets such as supermarkets [11] and fresh food stores [12] are more likely to have a healthier diet than those who live closer to unhealthy food outlets such as fast food restaurants [13, 14]. Socioeconomic disparities also affect population diet quality and can lead to inequalities in health outcomes [15–20]. The SES of a local area has been indicated as a determinant that shapes the local food retail environment, thereby impacting population dietary behaviours and rates of obesity and cardiovascular disease [21–23]. Food acquisition behaviours, such as grocery shopping, are also influenced by the community food environment [24, 25]. Associations have been shown between home food inventory and diet quality in both children [26, 27] and adults [28, 29]. However, a systematic review conducted in 2015 found limited evidence on the associations between obesity and the local food environment [30]. To date, most studies have been conducted in the western context, and there is very limited research conducted in the East Asia and Pacific regions where the food, political and cultural climates are vastly different.

Within these Asian countries, Hong Kong and Singapore were selected as they uniquely share similar economic, environmental and population characteristics. Both are high-income countries with well developed economies [2, 31] focused on services (tertiary sector) with minimal domestic production and manufacturing. Both countries have high population and food outlet densities, limited land space, multicultural societies, and heavily rely on food import [32–34], contributing to a unique food landscape considerably influenced by globalisation. The selection of Hong Kong and Singapore allowed for comparison of the food environment to be made across these two countries whilst allowing for a bridge with the knowledge generated on food environments within the western context.

Singapore and Hong Kong import 90% and 95% of goods in the food and beverage market respectively [35–37]. Consequently, retail prices of food in these countries are highly dependent on the global food market [30, 38]. Due to these factors, Hong Kong and Singapore share similar but unique food environments and cultures [39, 40] which distinguish them from the western countries in which most of the current available data are based on. This makes them suitable as a case study to explore the food environment in developed Asian countries in the context of differing socioeconomic neighbourhoods, to inform effective and cultural-specific policy influencing population health.

Both Hong Kong and Singapore experience income disparities despite relatively high national gross domestic products (GDP) per capita [41, 42]. In context, Hong Kong reports a Gini coefficient of 0.533 [43] (a measure of the income distribution of the country with a higher number indicating higher inequality), which is among the highest values for a developed country. Singapore reports a Gini coefficient of 0.345 [44]. Evidence indicates that there are multiple factors which contribute to dietary inequalities between socioeconomic positions in Hong Kong and Singapore. A report released by WHO cited that a healthy diet, a diet that provides adequate calories and nutrients including a diverse food choices from a range of food groups, costs up to approximately 4 times more than that of an energy sufficient diet, a diet which only provides adequate calories, in Hong Kong [2]. A recent study conducted in China on the link between population dietary knowledge, SES, and stroke in adults discovered that higher dietary knowledge was associated with a lower risk of stroke, while SES was a significant factor in predicting dietary knowledge [45]. To date, there is currently scarce research exploring the association between the spatial arrangement of food outlets and SES in both countries. Thus, further evaluation of the differences in the food environment of high and low SES areas is needed to better understand and address the local health disparities in both Hong Kong and Singapore.

Government efforts in Singapore throughout the last decade to improve the food retail environment have been directed mainly towards promoting the use of healthier ingredients as opposed to managing the placement and accessibility of various food outlets [46]. A prominent program is the Health Promotion Board (HPB)’s Healthier Dining Programme, where food outlets are encouraged to source healthier ingredients from suppliers and are rewarded for providing and promoting healthier options to consumers. Some incentives include financial grants and increased publicity through a separate healthy living government campaign [46]. Even with the implementation of such programs, the prevalence of chronic diseases remains elevated and some diet-related preventable conditions, such as type 2 diabetes mellitus, were found to have increased in 2020 in Singapore [47]. This gives cause for the exploration and introduction of complementary strategies to boost the efficacy of current interventions in cultivating a healthier food environment. As a small country, the limited land resources in Singapore pose further challenges to city planning, as evidenced by the current lack of robust local government policies and zoning laws implemented for food outlets [48].

Policies in Hong Kong aimed at reducing the prevalence of non-communicable diseases among the population have been implemented with nine goals to be achieved by 2025 [49]. These policies involve a range of evidence-based strategies including but not limited to ingredient bans, media campaigns, subsidies, labeling strategies and education. Current policy efforts relating to public health nutrition are in early development compared to those in western countries [49]. Ongoing efforts are largely centered around mass media campaigns to raise population awareness [49]. Limited policy effort surrounding the management and facilitation of a healthier food environment prompts an opportunity for research on the food retail environment specifically within the context of Hong Kong.

The aim of this paper is to explore the local food environment of Hong Kong and Singapore through the mapping and analysis of the placement and density of food outlets in selected high SES and low SES areas in order to identify differences which may influence population food options and choices. This evidence can be used to inform upstream policy making so as to improve long term population health from a nutrition perspective.

## Methods

A case study approach [50] was used. The following steps were taken: (1) Determination of socioeconomically advantaged/disadvantaged areas based on secondary data. (2) Definition and classification of the type of food retail outlets specific to these two countries, relevant to the procurement of healthy and unhealthy foods. (3) Digital searching and mapping of food outlets within the designated areas. (4) Physical data collection and confirmation of food outlets mapped digitally.

### Determination of socioeconomically advantaged and disadvantaged areas

To investigate the association between the placement and density of food outlets and SES within these two countries, socioeconomic disparities between suburbs were identified using secondary data on socioeconomic index and household income for suburbs within Singapore [51] and Hong Kong [34] respectively. The area that was chosen was based on the government's definition of districts. This was done because demographic data were available for these districts. In Singapore, the areas of Outram and Newton, suburbs ranked highest and lowest on the socioeconomically disadvantaged index (SEDI) respectively, were assessed [51]. In Hong Kong, the district with the highest median household income, Central and Western District, was compared with Sham Shui Po (SSP) District, the district with the lowest median household income [34].

### Definition and classification of food retail outlets

To ensure consistency in the classification of local food retail outlets, definitions and a classification system for food outlets spanning the two countries was developed. The suitability of the North American Industry Classification System (NAICS), a coding system used to define food outlets in the industry to allow for comparison between the US, Canada and Mexico, was considered [52]. We found that the NAICS system did not account for all food outlet types available in Hong Kong and Singapore. An instance of this is the absence of wet-markets from their system. These are generally not found in North America and therefore do not have a formal NAICS code; hence, there was a need to generate a classification system suitable for Singapore and Hong Kong.

A total of 18 food outlets were initially classified and defined by combining the NAICS list of food business types with the food outlet classification systems provided by the local government websites for both Hong Kong and Singapore [46, 52]. Descriptions of these food outlet types were compared between Hong Kong and Singapore to standardise definitions. Of these, a final 11 food outlet types were identified as those commonly frequented for groceries and take-away food options through consultation with a panel of 9 local residents, 3 from Singapore, 6 from Hong Kong, and selected for mapping to reflect where food is purchased for at-home eating (Table 1).

**Table 1:** Food outlet definition and classification derived from the Singapore and Hong Kong government websites and the NAICS (Health Promotion Board, 2021, NAICS Association, 2021)

Outlet	Definition
*Supermarkets*	Stores which sell a range of general line products, including but not limited to fresh fruits and vegetables, shelf-stable goods (canned, bottled, packaged), fresh/frozen meat, fish and poultry
*Farmer’s markets*	Single or a collection of vendors which primarily engage in selling of fresh produce items
*Wet markets/traditional markets*	A collection of vendors or areas which consist of selling fresh produce, meat/fish/poultry, cooked foods as well as clothing and household items
*Green grocers*	Shops with a permanent store space primarily retailing a variety of health food products and fruits and vegetables
*Convenience stores*	Small scale stores which retail primarily a range of packaged food and drink items. These stores may also serve simply prepared hot foods and usually little or no choice of fresh fruit/vegetable products
*Fast food outlets*	Restaurant establishments which provide food services, commonly consisting of ultra-processed food items, prioritising speed and efficiency. Patrons usually pay before eating and food can be consumed in-store, taken out, or delivered. The definition has been expanded to include Chinese fast-food chains (café de coral, Fairwood) which serves a variety of Chinese/western dishes at low price and often with poor nutrition qualities
*Take-aways/food stalls*	Permanent food establishments which mainly serve hot food and non-alcoholic drink items made to order. These establishments offer limited dine-in space (< 10 seats) and function mainly for customers to takeaway food
*Bakeries*	Stores which sell confectionery and baked primarily. Products may be made in-store or as a manufacturer retail format and offer no in-store seating. Sitting areas may or may not be available. The sale of beverages may or may not be included
*Butchers*	Stores/vendors that are separate from a collection of stores (public markets) which sells fresh/frozen/cured meat and poultry products
*Breakfast shops*	Breakfast shops are places where foods are made by order, and some ready made items are sold, such as sandwiches, bottled drinks. The opening hour of breakfast shops is usually from 4am to 1 pm. Some breakfast shops offer spaces to dine in, but with limited seats (< 20 seats). Breakfast shops are defined differently from take-aways/food stalls as they have distinct opening hours
*Food courts/food gourmets*	A common dining area with access to multiple food and beverage stalls. Customers have to self-service purchase of food. Food courts can be indoors or outdoors. If indoors, typically located in malls, hospitals and buildings, etc. If outdoors, typically called hawker centres

The study excluded seven types of food outlets from its analysis, namely restaurants, cafes, pubs/bars, canteens, cooking studios, drink shops, and night markets. These outlets were considered primarily as dine-in options and were not directly linked to food procurement that affects home diet, as agreed upon by the panel of local researchers.

The 11 food outlet types selected for mapping include bakeries; take-aways/food stalls; food courts/food gourmets; breakfast shops; supermarkets; convenience stores; farmer’s markets; wet markets/traditional markets; butchers; greengrocers; fast food outlets. Fast food outlets were included in the mapping of the Hong Kong suburbs but not in Singapore as studies by Zhang et al. and Cheung et al.found that unhealthy food outlets in Hong Kong are often strategically placed near vulnerable groups, such as within walking distance from secondary schools [53, 54]. On the other hand, hawker centres, defined as food courts located outdoors (classified as food courts in this study), were exclusive to Singapore’s mapping as research has highlighted hawker centres as one of the most integral parts of Singapore food culture [55, 56]. In a qualitative study by Lim et al. in Singapore, 45% of participants reported dining from hawker centres 3–7 days of the week [55]. Additionally, Foo et al. found that half of the Singapore population frequent hawker centres at least once a day [56].

### Mapping procedure of food outlets

Geographical mapping of the various food retail types was conducted in two phases. In both countries, each type of food outlet in the selected suburbs was quantified and first mapped digitally through Google Maps [57], compiled, then labelled using a standardised colour-code.

In the higher SES district of Singapore, Newton, the defined district area had minimal food outlets and the mapped area was extended for a more reflective picture of the food environment as the neighbouring district had numerous shopping centres that acted as a food hub within the area.

In the case of Hong Kong, an additional map of food outlets located within a 500 m radius, equivalent to a 5–10-min walk, of the Mass Transit Railway (MTR) station in each area was conducted. With the MTR being Hong Kong’s primary mode of public transport, food outlets are concentrated within the immediate area surrounding the station. These areas experience the highest traffic and mapping of the food outlets in these areas may provide a more accurate representation of the food environment in each district. The 500 m radius was determined by Euclidean distance and was similarly mapped using the Google MyMaps software [57].

Researchers local to each country employed the ground-truthing method in addition to digital mapping to ensure that the mapping was both comprehensive and reflective of the actual food environments. This involved a final step of cross-referencing on-foot for the 500 m areas in each district of Hong Kong and the full district areas in Singapore. On-foot cross-referencing could not be conducted on the full district maps for Hong Kong due to limited resources as these districts covered a much larger land area than Singapore’s districts.

### Data collection

Teams of three to six researchers, led by researchers IH and TChng, were briefed on the protocol prior to the commencement of data collection and mapping of the food outlets in Singapore and Hong Kong respectively. Both teams were comprised of local researchers familiar with the food and social cultures in each respective country. Data collection took place from February 2021 to April 2021 and data was stored on a Google Drive folder which has been made publicly accessible (in availability of data and materials). Individual and organisational information were not collected as part of the research, hence ethics approval and consent to participate were not applicable as no humans were involved and only publicly available data were used.

### Data analysis

Food outlet density was determined by dividing the total number of food outlets by the land area and was completed for both districts in Singapore as well as the 500 m radius around the main MTR station of both Hong Kong districts. District population data were collected from the government websites of both countries to determine the food outlet density of each area per every 1000 residents.

Exclusive to the study conducted in Hong Kong, the relative proportion of unhealthy outlets to total outlets within the district-wide area was determined using a modified retail food environment index (mFREI). Food outlets were classified as healthy and unhealthy outlets, in order to align with the study by Zhang et al.(2018) to allow for suitable comparison [53]. It is to be noted that the food outlet classification was slightly different to the one used by Zhang et al. due to the necessity for our list to be specific to both Hong Kong and Singapore. Places which predominantly sell fresh food ingredients (supermarkets, wet markets and farmer’s markets) were classified as healthy outlets whilst stores which sell convenience and/or fast foods (convenience and fast-food restaurants) were classified as unhealthy outlets. All other food outlets which did not fall into these categories were included in the ‘others’ section; these included bakeries, take-away stalls, food courts/canteens, breakfast shops, butchers and greengrocers. The modified retail food environment index (mRFEI) was calculated using the indicator adapted from Mahendra et al.(2017) for the districts of Hong Kong. The mRFEI is the total number of unhealthy outlets within an area divided by the total number of healthy and unhealthy outlets within the area combined. A higher score indicates a less healthy food environment [58]. As the equation was originally developed to be used in the setting of Canada where wet markets are scarce, wet markets were included as a healthy food outlet in the calculations to provide a more accurate comparison when used in the setting of Asian countries to align with the methodology by Zhang et al*.* (2018) [53]. This methodology was not replicated in Singapore due to limitation of human resources during the study period.

## Results

### Hong Kong

#### 500 m radius surrounding the station

##### Sham Shui Po station (Low SES)

**Fig. 1:**
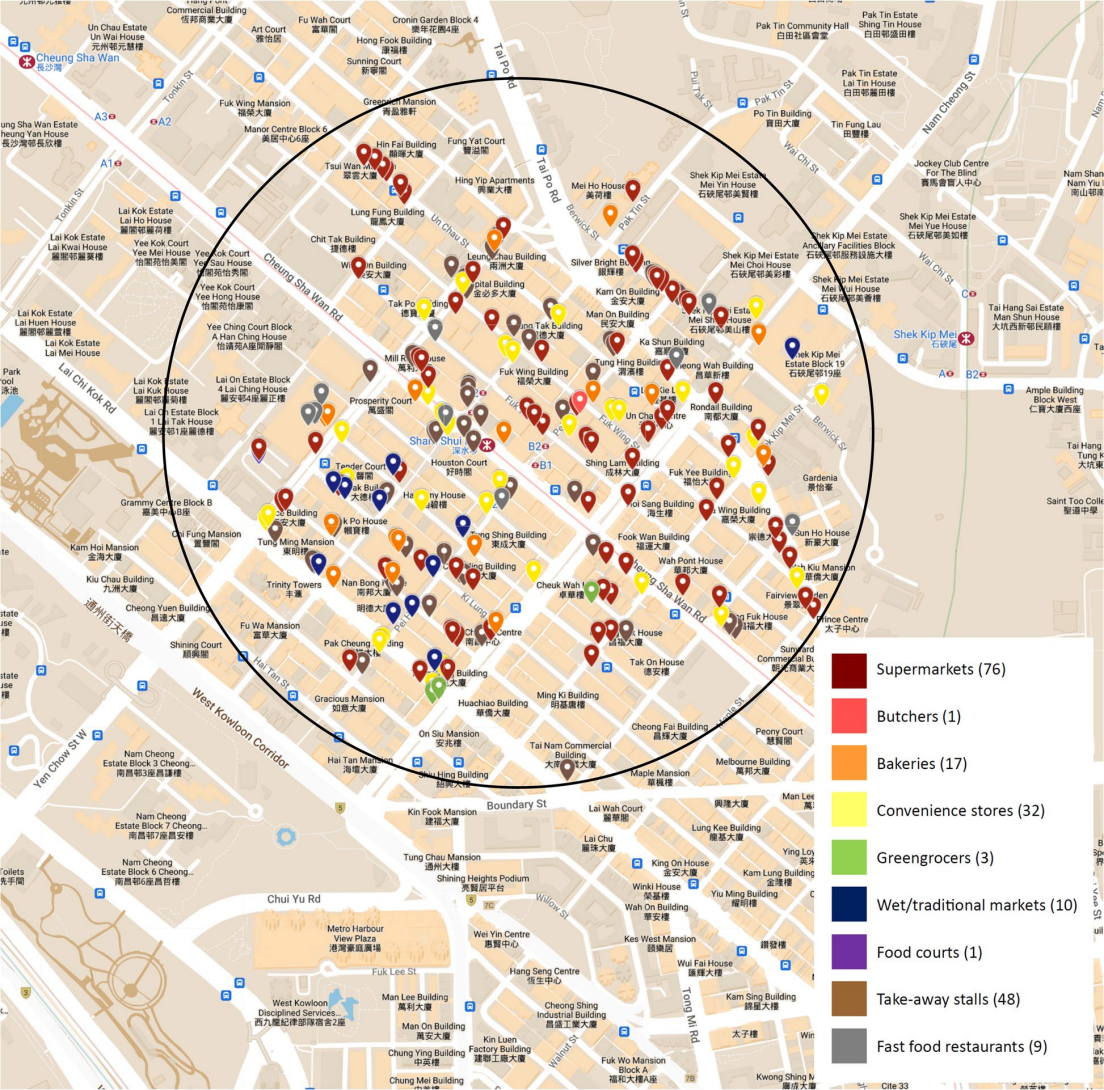
Food outlets within 500 m radius of Sham Shui Po station, within Sham Shui Po District in Hong Kong

A total of 197 food outlets were mapped within the 500 m radius surrounding the SSP station (Fig. 1), with supermarkets being the most commonly found outlet type (76 or 38.6%). Other food outlets mapped consisted of 48 take-away outlets (24.4%), 32 convenience stores (16.2%), 17 bakeries (8.6%), 10 wet markets (5.1%), nine fast food outlets (4.6%), three greengrocers (1.5%), one food court and one butcher shop (0.5% each). As shown in Table 2, a food outlet density of 250.8 outlets per km^2^ was estimated in SSP, with food outlets distributed on almost all streets surveyed. Comparing the healthiness of the food outlets found, healthy food outlets made up 43.7% of the area’s food outlets and unhealthy outlets contributed to 20.8%, and other outlets making the remaining 35.5%. There was a high number of small independent supermarkets observed, which contributed to a high percentage of healthy food outlets.

**Table 2:** Food environment indicators for the 500 m radius area surrounding Central station (within Central and Western District) and Sham Shui Po station (within Sham Shui Po District) in Hong Kong

Indicators	Central Station (high SES)	Sham Shui Po Station (low SES)
Number of food outlets	*n* = 102	*n* = 197
Healthy food outlets (Supermarkets, wet markets)	18 (17.6%)	86 (43.7%)
Unhealthy food outlets (fast food outlets, convenient stores)	28 (27.5%)	41 (20.8%)
Other food outlets (bakeries, take-away stalls)	56 (54.9%)	70 (35.5%)
Food outlet density (outlet/km^2^)	129.9	250.8

##### Central station (High SES)

**Fig. 2:**
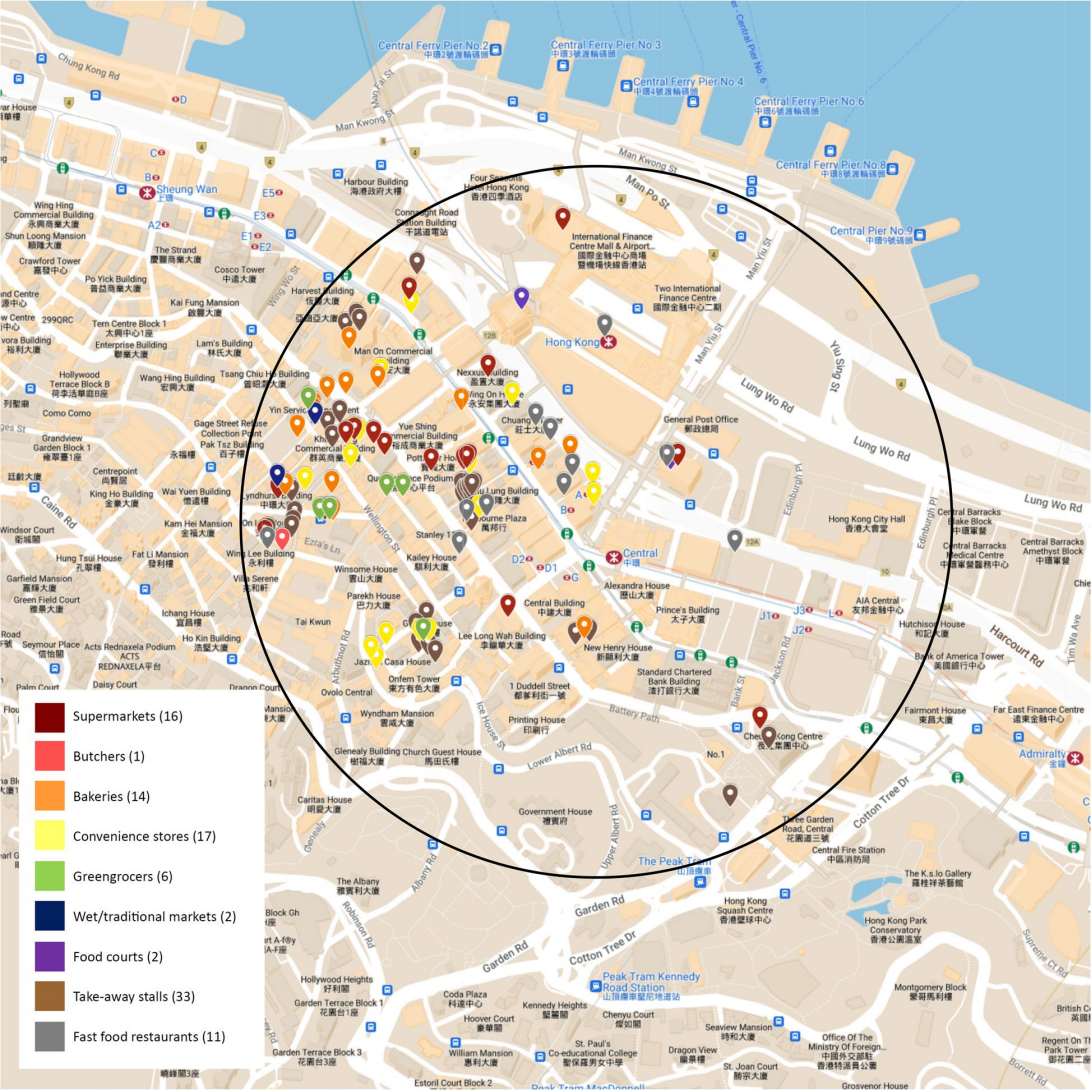
Food outlets within 500 m radius of Central station, Central and Western District in Hong Kong

A total of 102 food outlets were mapped in the 500 m radius surrounding Central station, (Fig. 2), with the most common food outlet category being take-away outlets at 33 (32.3%). Other food outlets mapped in the area include 17 convenience stores (16.7%), 16 supermarkets (15.7%), 14 bakeries (13.7%), 11 fast food outlets (10.8%), six greengrocers (5.8%), two food courts and two wet markets (2.0% each), as well as one butcher shop (1.0%). The food outlet density in this area was 129.9 outlets per km^2^ (Table 2), with the majority of food outlets distributed west of the station, located near residential housings. Conversely, there was a lack of food outlets towards the south and east of the station with commercial and government establishments taking up these areas. Healthy food outlets made up 17.6%, unhealthy food outlets made up 27.5% of the total outlets in this area and food outlets classified under ‘others’ made up the remaining 54.9%.

#### The greater district area

##### Sham Shui Po (Low SES)

**Fig. 3:**
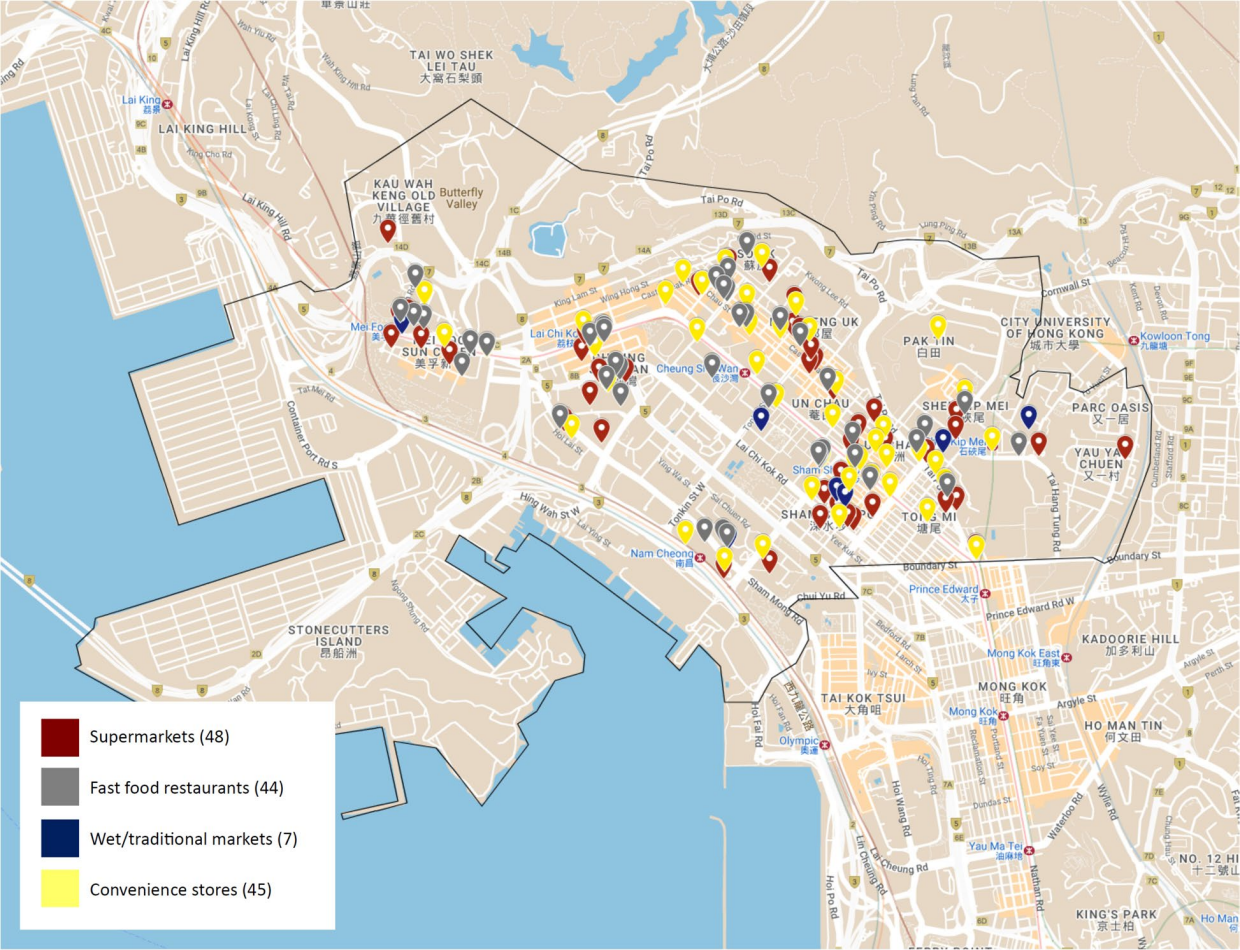
Food outlets located in Sham Shui Po District of Hong Kong

A total of 144 food outlets were mapped digitally in the greater SSP district (Fig. 3); consisting of 48 supermarkets (33.3%), 45 convenience stores (31.3%), 44 fast food outlets (30.6%), and seven wet markets (4.9%). With SSP being a largely residential district, food outlets were distributed relatively evenly throughout; the only exception being the cargo bay area towards the west of the district where factories and storage houses are predominantly located. The total district area of the area was 9.48 km^2^, and the area was found to have a food outlet density of 15.2 outlets per km^2^ (Table 3). Healthy outlets made up 38.2% of the total outlets whilst unhealthy outlets made up 61.8% of mapped locations (Table 3).

**Table 3:** Food outlet data in Central and Western District and Sham Shui Po District in Hong Kong

Indicators	District	
	Central and Western District	Sham Shui Po District
Total number of food outlets	*n* = 202	*n* = 144
Healthy outlets	71 (35.1%)	55 (38.2%)
Unhealthy outlets	131 (64.9%)	89 (61.8%)
Food outlet density (outlets/km^2^)	16.1	15.2
Food outlet per 1000 residents	0.20	0.14
Food outlets per 1000 residents per km^2^	0.02	0.02
mRFEI	0.65	0.62

##### Central and Western District (High SES)

**Fig. 4:**
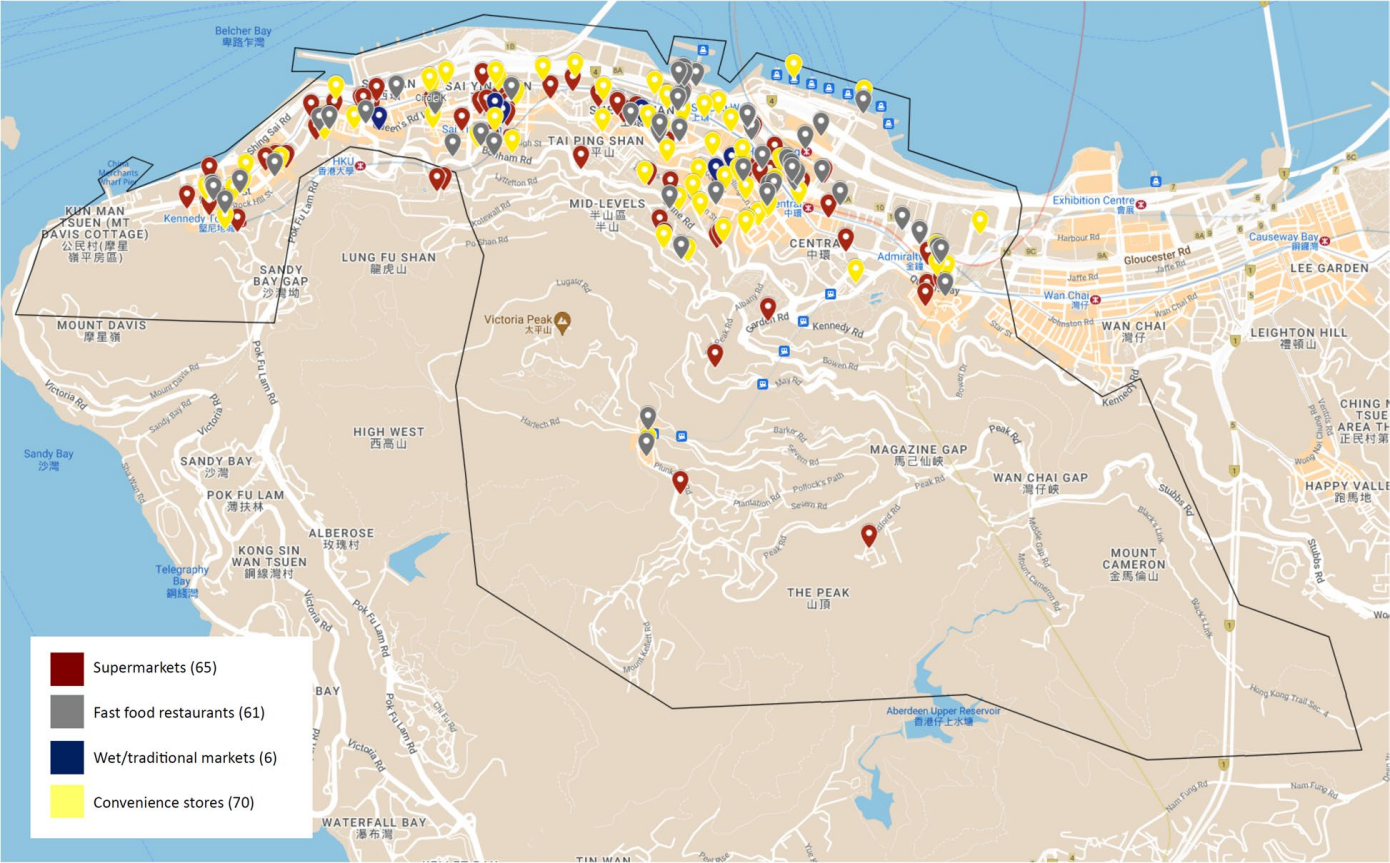
Food outlets located in Central and Western District of Hong Kong

In the Central and Western District (Fig. 4), a total of 202 food outlets were mapped digitally. This consisted of 70 convenience stores (34.7%), 65 supermarkets (32.2%), 61 fast food outlets (30.1%) and six wet markets (3.0%). Food outlets were largely distributed near the northern coast line where the stations of the mass transit railway extend from Central station in the east to Kennedy Town Station in the west. Residential areas predominantly make up the southern regions of this district, with mountainous terrain and access mainly via cars and buses. Food outlets were few and far between in these areas. Healthy outlets made up 35.1% of the total food outlets, whilst unhealthy outlets made up the remaining 64.9% (Table 3).

Overall, both districts reported similar mRFEI measures; 0.65 for Central and Western District and 0.62 for SSP District, indicating that the proportion of healthy and unhealthy food outlets in each district was similar; 35.1% and 64.9% versus 39.2% and 61.8% respectively (Table 3).

### Singapore

#### Newton

**Fig. 5:**
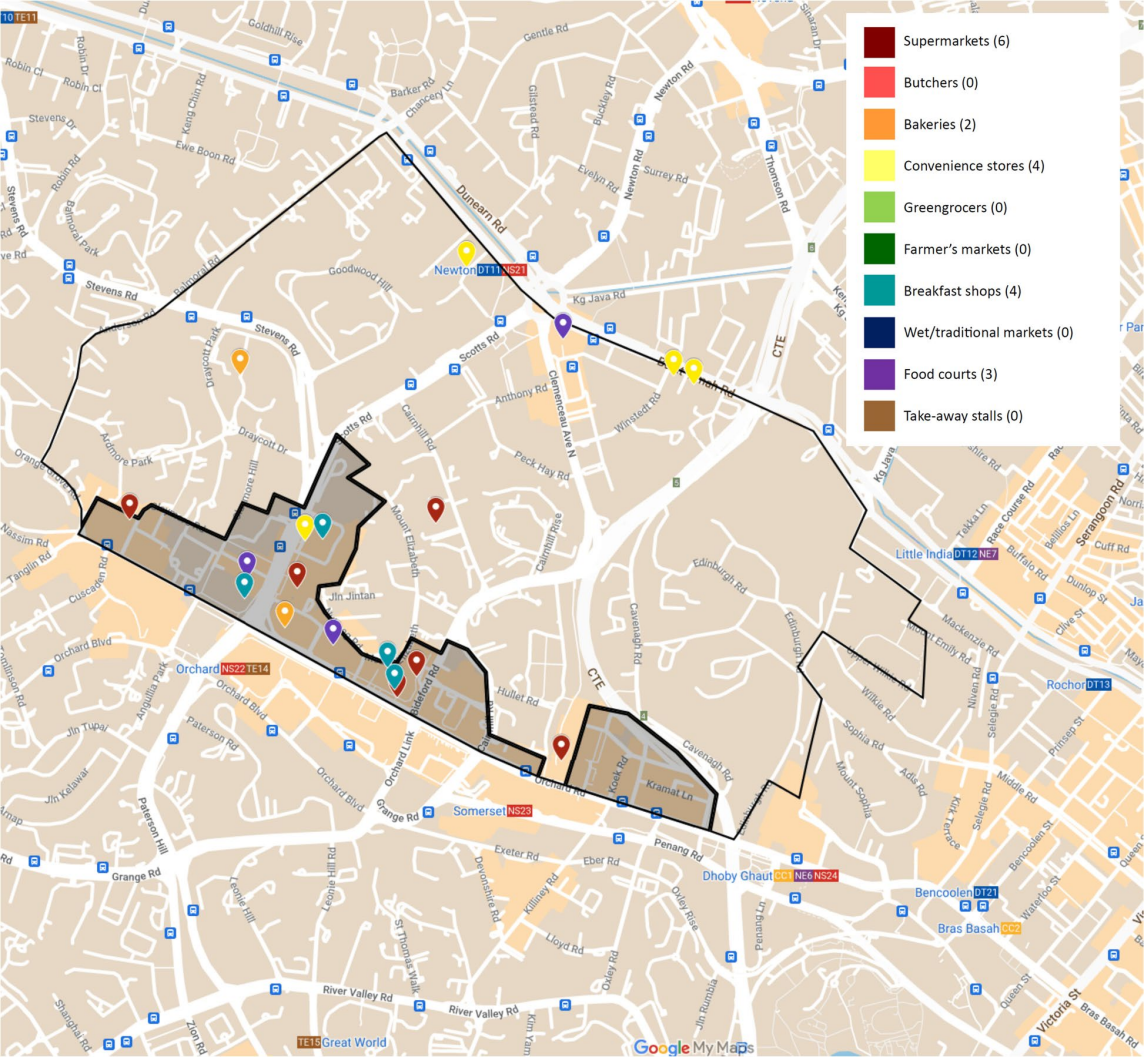
Food outlets in and the immediate area outside of the Newton area of Singapore

Within the Newton district (non-shaded enclosed area in Fig. 5), a total of seven food retail outlets were identified, three being convenience stores (15.8%), alongside one supermarket (5.3%), one bakery (5.3%), and one food court (5.3%). The extension into the neighbouring area (shaded enclosed area in Fig. 5) had shopping centres that housed more food retail outlets – five supermarkets (26.3%), four breakfast shops (21.1%), two food courts (10.5%), one convenience store (5.3%) and one bakery (5.3%). Even with the extended area, there were no butchers, greengrocers, farmer’s markets, wet/traditional markets and take-away food stalls identified. With a district area of 2.07km^2^, Newton had a food outlet density of 9.2 outlets/km^2^ (Table 4).

**Table 4:** Comparison of food outlet data in Newton and Outram District in Singapore, district land areas^a^

Indicators	District	
	Newton	Outram
Total number of food outlets	19	95
District Area (km^2^)	2.1	1.4
Food outlet density (outlets/km^2^)	9.2	69.3
District Population	8260	18,340
Food outlet per 1000 residences	2.3	5.2
Food outlet per 1000 person per km^2^	1.1	3.8

^a^Citypopulation.de, 2020 [59]

#### Outram

**Fig. 6:**
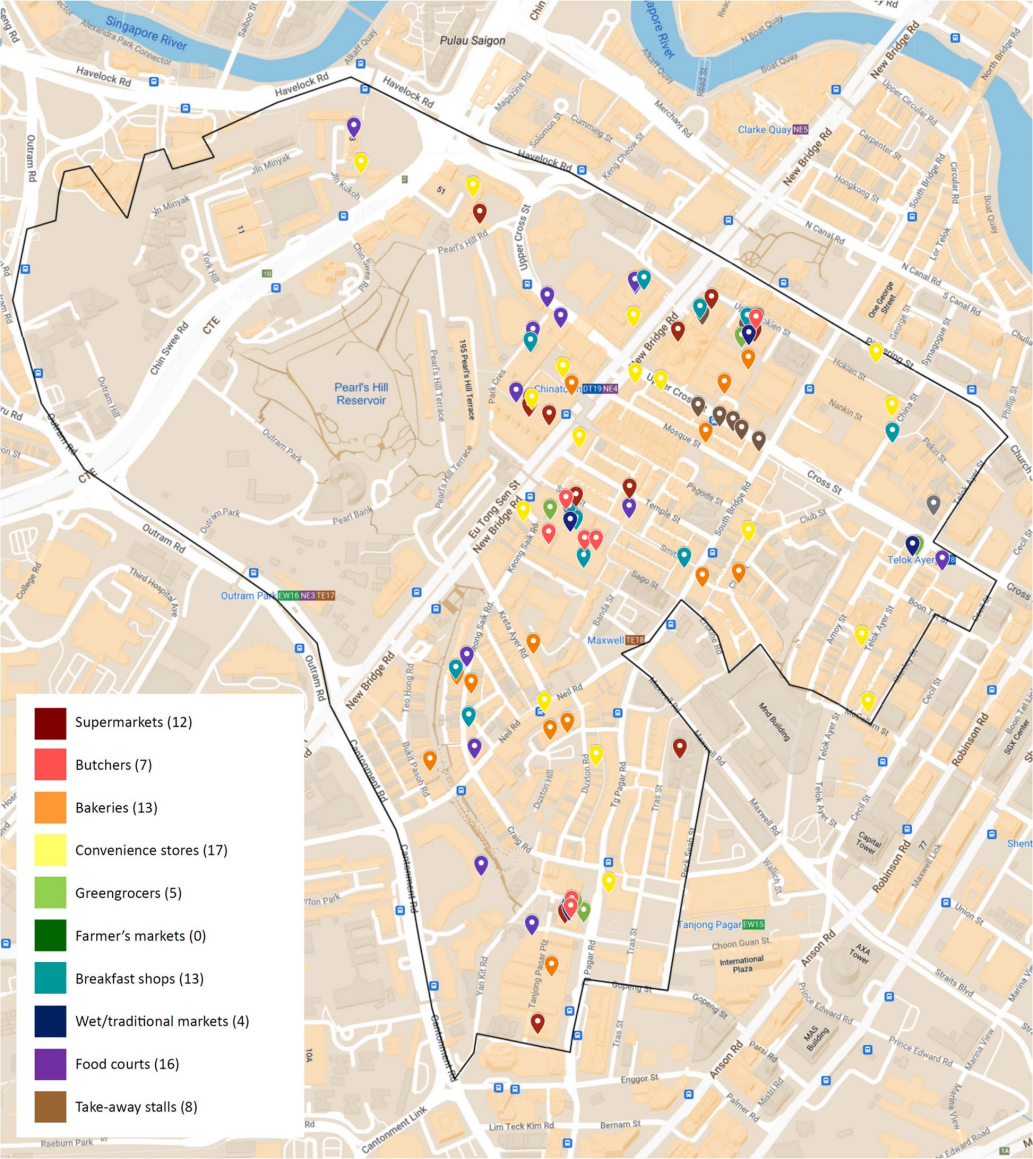
Food outlets in the Outram area of Singapore

In Outram, a total of 95 food outlets were mapped (Fig. 6); of which the most common food retail outlets were convenience stores (17.9%) and food courts (16.8%), with 17 and 16 outlets mapped respectively. There were 12 supermarkets (12.6%), 13 bakeries (13.7%) and 13 breakfast shops (13.7%) in the area. Some food courts had an attached wet/traditional market in the same location where fresh produce was sold, but there was a lack of farmer’s markets in the area. Most food outlets were gathered along public transport routes such as bus stops and the Chinatown train station but dispersed relatively evenly across the Outram area. With a district area of 1.37km^2^, Outram had a food outlet density of 69.3 outlets/km^2^ (Table 4).

## Discussion

This investigation of differences in food outlet types and their density by high and low SES areas reveals that in both Hong Kong and Singapore, lower SES areas surveyed were shown to have higher food outlet density while higher SES areas had fewer but larger food outlets. In Hong Kong, both SES areas reported similar proportions of healthy and unhealthy food outlets.

We observed a notable difference in the food outlet density between different SES areas of Singapore, whilst in Hong Kong, the food outlet density was similar when comparing between districts. Looking at the immediate area surrounding the stations of each area, the same trend can be observed; the food outlet density in the lower socioeconomic area is nearly double of the high socioeconomic area. This may be more telling of the local food environment considering a cargo pier and mountainous terrain make up a good proportion of the district landscape for the Central and Western and SSP districts respectively of Hong Kong. Multiple factors may have contributed to the differences in food environments between the differing SES areas of each country. Considering the lack of zoning laws in place for each country regarding the distribution of food retail outlets, it is likely that the clustering of food outlets near high traffic areas and residential areas is driven by economic viability, wherein the supply and distribution of food outlets in a certain area typically reflect the population density and consequent demand accordingly.

We found that in the 500 m radius surrounding the stations in Hong Kong, there was a much larger number of supermarkets, contributing to a higher proportion of healthy outlets when compared to the higher SES area of Central. This higher quantity of stores did not account for the size of these outlets, which in most cases observed, were typically smaller in the lower SES area of SSP. Further investigation is needed to understand the effects of size, number and capacity of food outlets and how these influence consumer purchasing behaviour and subsequently their health.

Recent research has shed light into the link between globalisation and diet [60, 61]. Although there is mixed evidence on the association between globalisation and diet outcomes, our findings suggest that SES, in the case of Hong Kong and Singapore, is a factor which may influence this association. Future research is needed to investigate the impact of these differences in SES and the food environment on diet quality to better target policies which can mediate obesity and chronic disease incidence in Hong Kong and Singapore.

Comparing the modified retail food environment index (mRFEI) between the two areas in Hong Kong, the ratio of healthy and unhealthy food outlets appeared to be similar between the two socioeconomic areas. Although the mRFEI was not utilised for comparison between the Singapore districts, given the small land area of both countries and taking into account the different types of transportations available, a lack of access to fresh healthy food may not be a primary issue. Food deserts, which are areas where residents lack access to healthy food outlets, have been found as a major predictor of poor population health in studies conducted in the US [62, 63]. Whereas, food swamps, areas in which residents are exposed to a high number of unhealthy food outlets, have been proposed to stimulate less healthy food choices and encourage the prioritisation of instant-gratification over long term health [64].

The findings of this study suggest that food swamps may have a bigger impact on population diet and health in the settings of Hong Kong and Singapore than food deserts. Zhang and Huang (2018) reported food swamps to be positively associated with poorer fruit and vegetable consumption and higher obesity rates in Hong Kong [53]. Cooksey-Stowers et al. (2017) found that the food swamp effect had a greater effect than food deserts in US counties with greater income inequality as well as where residents were less mobile [64]. In a culture which prioritises efficiency, convenience and eating out, as that in Hong Kong and Singapore, high accessibility to unhealthy take-away outlets and hawker centres in lower SES areas may negatively affect diet quality; especially when the costs of such take-away options are low [2, 48].

Transportation is a major determinant of the ability and distance that residents can or will travel to acquire food. It is likely that high income households typically have greater access to private transportation and are more likely to be able and willing to travel further outside their district to food outlets in both Hong Kong and Singapore. Consideration must however be given to the difference between Hong Kong, Singapore and Western countries regarding the extent of development of the public transport system. Both Hong Kong and Singapore have highly robust public transport systems with high modal shares [65]. Caution must therefore be taken when attempting to translate conclusions from studies conducted in western countries to the setting of Hong Kong and Singapore [66, 67]. To the researchers’ knowledge, there is no current evidence documenting the influence of public transport versus private transport on food acquisition behaviours specific to the context of Hong Kong or Singapore. This may be an area for further investigation.

Whilst it is no easy task to set up policies intending to influence a food environment driven by the private sector developed through supply and demand, targeted intervention in these areas may have a larger influence in the overall food environment in the case of Hong Kong and Singapore. Current research shows the importance of aligning the policy goals with stakeholder goals, in particular, alignment with financial goals in the private sector is required to maximise involvement and enhance the outcome [68]. Co-production has garnered a lot of attention recently in the field of public health policy making, with the benefit of being to better address barriers by drawing from the expertise of various stakeholders [69]. In the case of Hong Kong and Singapore, the large number of individual businesses run by individuals, families and companies with different motivations may make it difficult when trying to align the goals of each party during the process of co-production, causing delays in decision-making and strategy development. More research is necessary to assess the efficacy of these policy-making approaches in areas of Hong Kong and Singapore where food outlet density is much higher than western countries.

When assessing the home diet of high-income Asian countries such as Singapore and Hong Kong, it is worthwhile to consider the role and prevalence of domestic helpers. Migrant domestic workers (MDW) refer to any person moving to another country to improve their material or social conditions and the prospect for their families [70] by engaging in domestic work within an employment relationship [71]. Currently, Hong Kong and Singapore employ 340 and 250 thousand MDW respectively [72, 73]. MDW tends to be employed in higher income households to support home and child caring. One of the main responsibilities of MDW employed in households is the preparation of meals. This may also entail shopping for groceries. While those of higher socioeconomic positions typically rely on private transport as mentioned above, the MDW employed in these households often do not. MDW often rely on public transport or travel to food outlets by foot. This could conversely increase the demand for accessible grocery outlets within higher SES residential areas and should be considered in the development of related upstream policies such as zoning laws for food outlets. As such, the prevalence of MDW employment in higher income households offer an additional point of consideration when evaluating the relationship between the food environment and shopping behaviours in differing SES areas. This prevalence of MDW employment is a unique point of difference from western settings, and thus must be taken into account when extrapolating findings from other studies to settings similar to Hong Kong and Singapore.

## Strengths

This study was undertaken by respective teams based in-country, as part of the fulfilment of a 7-week full time Monash University Masters of Dietetics public health nutrition placement, in Singapore and Hong Kong. Each team was composed of local researchers familiar with the local food and social culture of their respective countries. This enabled the selection and mapping of country-relevant food outlets key to the local food environment. Notably, fast food outlets were mapped in Hong Kong following recent studies highlighting the negative health consequences related to the rising number of fast food outlets in Hong Kong, while hawker centres were included as food courts in the Singapore food outlet maps due to their pivotal role in the Singapore food culture.

Currently, there is limited available evidence on the geographical distribution of food outlets across Singapore and Hong Kong. As the first of its kind, this paper provides the foundation and direction for further research in this area. Future studies investigating a greater range of food outlets or analysing the food environment in other Asian countries of varying backgrounds (income, land area, population density, reliance on food imports) could be useful for comparison with results obtained from this study. The consumer food environment also plays an important role in determining the food choices of consumers. Factors such as marketing and affordability including food pricing, placement, promotion and place, can influence what people purchase and eat [74, 75]. Deeper analysis on the quantity and distribution of food outlets providing healthy versus unhealthy menu options can provide further insight into the management of chronic disease and obesity from a public health perspective. This remains an area of research to be considered in conjunction with the community nutrition environment of the two countries depicted in this study.

## Limitations

Several limitations to this study should be considered. Firstly, although the two countries share multiple similarities and histories as described above, there remain many factors which make each country and culture distinct from one another, influencing the makeup of their food environment. Political, cultural, economic and geographic factors all contribute to how the food environment has evolved and will continue to change in the future and must be considered when interpreting findings.

The cross-sectional nature of this study may not be reflective of the rapidly changing food environment known in these two countries and does not allow for causal relationships to be established. Due to the small sample size of countries and districts assessed as a result of time and human resource constraints, statistical tests were not conducted to examine the statistical significance of reported differences.

At the time this study was conducted, the COVID-19 pandemic had not peaked in Singapore and Hong Kong and there were no heavy restrictions, such as forced closures in place, that significantly hindered data collection. While COVID-19 may have influenced public health perception and subsequent food procurement behaviours, the rapidly changing human and political environment during the study period made the impacts of these factors difficult to evaluate. Our study focussed on the geographical mapping and density of food outlets, independent to operating business hours, with the assumption that the food environment is still minimally affected by the COVID-19 pandemic.

The districts for this study were selected from secondary data on socioeconomic status and household income in Singapore and Hong Kong published in 2015 and 2019 respectively. In these countries, where the food environment and populations experience consistent, rapid changes, the sampling frame used may not be up to date or representative of the current socioeconomic standing of the districts in each country and any discrepancies may have been amplified based on choosing the two districts on the reported extremes of the socioeconomic spectrum. Future research should minimise this bias by adopting a more complete sampling frame with a range of districts across the socioeconomic spectrum and/or by expanding the area of study to encompass multiple districts in both countries. Furthermore, the digital mapping via GoogleMaps may not be a comprehensive picture of all the food outlets district-wide in which ground-truthing methods revealed food outlets which were not found digitally in the case of Hong Kong and Singapore. Although resource intensive, ground-truthing methods may prove to provide a more accurate representation of the local food environment.

Although a reclassification of the food outlet types was established, the generalisability of these definitions may be limited, especially when compared with countries that have significantly different demographic and cultural backgrounds.

Lastly, the food environment surrounding the residents’ home diet only reflects a portion of the food environment they are exposed to during the day, and thus may not accurately depict the overall food environment, especially in countries like Singapore and Hong Kong where there is a culture of eating out regularly. Thus, the cross-sectional nature of the study, small sample size and potential selection bias should be taken into consideration when interpreting the findings and making associations between the food environment and population diet.

## Conclusion

To our knowledge, this is the first study to compare the food environment across differing SES areas in Hong Kong and Singapore and to provide insight on the access to healthy food outlets using geographic mapping methods. This study examined the variation in types of food outlet and their densities among differing local SES areas. There was a higher number of food outlets in lower SES areas compared with the higher SES area in both countries. This may serve as a barrier in implementing food laws due to the high number of stakeholders. As the first of its kind, this study should be used to inform future relevant upstream public nutrition policies, such as zoning laws for food outlets, to strive for a better health-enabling food culture. Mapping methodology in future studies should consider utilising the ground-truthing method in conjunction with digital mapping, to provide a more comprehensive picture of the food environment. Our findings highlight the necessity for further region and country-specific research to avoid making incorrect assumptions and generalisations from the existing literature on food mapping, which has predominantly focused on Western nations, compared to Asian countries that may have distinctly different food environments. Consideration of the differences in food culture between areas as well as varying socioeconomic position and addressing the barriers specific to these environments is key for the development of effective and sustainable strategies to improve the food environment and long-term population health in the setting of urbanised Asian countries such as Hong Kong and Singapore.

## Supplementary Information

**Additional file 1.**

**Additional file 2.**

**Additional file 3.**

**Additional file 4.**

## Data Availability

Any restrictions to use by non-academics: Not applicable The dataset(s) supporting the conclusions of this article is available in the Google Maps LLC Software repository, provided in the following hyperlinks: Singapore maps Outram:https://www.google.com/maps/d/u/1/edit?mid=1PsGCnvxzKGl9PQ8K7yh0LrGXsqcSnfSo&usp=sharing Newton: https://www.google.com/maps/d/u/1/edit?mid=1EssjypHKWnyhMApESVb4IJ2Z5g0D-RlU&usp=sharing Hong Kong maps District maps: https://www.google.com/maps/d/u/0/embed?mid=1fHtJTirQAZun_C4HWerryMvcmFBE3hUg&ehbc=2E312F 500m Radius maps: https://www.google.com/maps/d/u/0/embed?mid=14zC9e238lYpyxYpQZOJgaUFp1oXT8yrI&ehbc=2E312F Project name: Google MyMaps Project home page: https://www.google.com/mymaps Archived version: Refer to KMZ files provided in additional files submitted Operating system(s): Platform independent Programming language: Java, C++, XML Other requirements: Web browser: Google Chrome, Firefox, Microsoft Edge, Safari License: Google LLC.

## Abbreviations

FAO: Food and agriculture organisation
GDP: Gross domestic product
km: Kilometre
MDW: Migrant domestic workers
mRFEI: Modified retail food environment index
NAICS: North American industry classification system
SEDI: Socioeconomically disadvantaged index
SES: Socioeconomic status
SSP: Sham Shui Po *(District in Hong Kong)*
US: United States
WHO: World Health Organisation
